# Exotoxin S secreted by internalized *Pseudomonas aeruginosa* delays lytic host cell death

**DOI:** 10.1371/journal.ppat.1010306

**Published:** 2022-02-07

**Authors:** Abby R. Kroken, Naren Gajenthra Kumar, Timothy L. Yahr, Benjamin E. Smith, Vincent Nieto, Hart Horneman, David J. Evans, Suzanne M. J. Fleiszig

**Affiliations:** 1 School of Optometry, University of California, Berkeley, Berkeley, California, United States of America; 2 Department of Microbiology and Immunology, Loyola University Chicago, Maywood, Illinois, United States of America; 3 Department of Microbiology and Immunology, University of Iowa, Iowa City, Iowa, United States of America; 4 Vision Science Program, University of California, Berkeley, Berkeley, California, United States of America; 5 College of Pharmacy, Touro University California, Vallejo, California, United States of America; 6 Graduate Groups in Microbiology, and Infectious Diseases & Immunity, University of California, Berkeley, Berkeley, California, United States of America; Children’s Hospital Boston, UNITED STATES

## Abstract

The *Pseudomonas aeruginosa* toxin ExoS, secreted by the type III secretion system (T3SS), supports intracellular persistence *via* its ADP-ribosyltransferase (ADPr) activity. For epithelial cells, this involves inhibiting vacuole acidification, promoting vacuolar escape, countering autophagy, and niche construction in the cytoplasm and within plasma membrane blebs. Paradoxically, ExoS and other *P*. *aeruginosa* T3SS effectors can also have antiphagocytic and cytotoxic activities. Here, we sought to reconcile these apparently contradictory activities of ExoS by studying the relationships between intracellular persistence and host epithelial cell death. Methods involved quantitative imaging and the use of antibiotics that vary in host cell membrane permeability to selectively kill intracellular and extracellular populations after invasion. Results showed that intracellular *P*. *aeruginosa* mutants lacking T3SS effector toxins could kill (permeabilize) cells when extracellular bacteria were eliminated. Surprisingly, wild-type strain PAO1 (encoding ExoS, ExoT and ExoY) caused cell death more slowly, the time extended from 5.2 to 9.5 h for corneal epithelial cells and from 10.2 to 13.0 h for HeLa cells. Use of specific mutants/complementation and controls for initial invasion showed that ExoS ADPr activity delayed cell death. Triggering T3SS expression only after bacteria invaded cells using rhamnose-induction in T3SS mutants rescued the ExoS-dependent intracellular phenotype, showing that injected effectors from extracellular bacteria were not required. The ADPr activity of ExoS was further found to support internalization by countering the antiphagocytic activity of both the ExoS and ExoT RhoGAP domains. Together, these results show two additional roles for ExoS ADPr activity in supporting the intracellular lifestyle of *P*. *aeruginosa*; suppression of host cell death to preserve a replicative niche and inhibition of T3SS effector antiphagocytic activities to allow invasion. These findings add to the growing body of evidence that ExoS-encoding (invasive) *P*. *aeruginosa* strains can be facultative intracellular pathogens, and that intracellularly secreted T3SS effectors contribute to pathogenesis.

## Author summary

While the ADPr domain of the T3SS effector ExoS plays multiple roles in the intracellular lifestyle of *P*. *aeruginosa*, ExoS can also be cytotoxic and/or antiphagocytic. Here, we show that when *P*. *aeruginosa* enters the cytosol of epithelial cells, cell death is triggered independently of T3SS effector toxins, but ExoS ADPr activity delays this to enable continued intracellular survival and replication. Using rhamnose induction to express the T3SS only after invasion restored this ExoS-dependent phenotype, showing that intracellularly secreted effectors can enable intracellular pathogenesis. ExoS ADPr activity also countered antiphagocytic activity of ExoS and ExoT RhoGAP domains. These results show two additional roles for ExoS ADPr activity in promoting internalization of *P*. *aeruginosa* and protecting the intracellular niche, continuing to challenge the notions that *P*. *aeruginosa* is exclusively an extracellular pathogen, that it needs to inject T3SS effectors across plasma membranes, and that ExoS is necessarily cytotoxic to host cells.

## Introduction

The intrinsic and acquired antibiotic resistances of the opportunistic pathogen *Pseudomonas aeruginosa* have led to its inclusion on the Center for Disease Control’s list of agents of serious concern, and classification as an ESKAPE pathogen of concern in nosocomial infections. While *P*. *aeruginosa* can infect a wide variety of host cell types, it most commonly targets compromised epithelia and phagocytes [1–4]. Key to acute infections caused by *P*. *aeruginosa* is its type III secretion system (T3SS) [5]. The T3SS apparatus functions as a molecular syringe to inject effector proteins directly into the host cell cytoplasm (reviewed in [6]). There are four characterized effectors in *P*. *aeruginosa*: ExoS, ExoT, ExoU and ExoY, with ExoS and ExoU generally being mutually exclusive [7]. While a majority of isolated strains encode the nucleotidyl cyclase ExoY, its effect on various types of host cells has only recently been explored, and it may play a role in limiting cytotoxicity elicited by other effectors [8]. ExoY can contribute to virulence in corneal cells [9], but appears dispensable in some *in vivo* models despite various *in vitro* phenotypes [10–13]. Strains encoding ExoU, a potent A2 phospholipase that causes rapid lysis [14,15], are appropriately designated “cytotoxic strains” and represent a minority of isolates [7,16]. Most *P*. *aeruginosa* isolates encode ExoS instead of ExoU; both clinical isolates and laboratory strains that encode ExoS display an invasive phenotype [16,17]. ExoT is almost universally encoded by *P*. *aeruginosa* strains [17].

ExoS and ExoT show 76% amino acid identity; both are bifunctional enzymes with an N-terminal RhoGTPase Activating Protein (RhoGAP) domain and a C-terminal ADP ribosyltransferase (ADPr) domain [18]. The RhoGAP domains of both effectors hydrolyze GTP to GDP in Rho, Rac, and Cdc42, leading to cytoskeletal depolymerization, which can reduce the capacity of host cells to phagocytose *P*. *aeruginosa* [18–21]. The targets of ExoS and ExoT ADPr activity are different, with ExoT targeting arginine residues of Crk1 and Crk2, which can cause focal adhesion disassembly [22], and ExoS having broad ADPr substrate specificity [18]. When injected across the plasma membrane, ExoS ADPr activity targets small GTPases: Ras, Rap1, Rab proteins (Rab 5, 7, 8, 11) to limit endocytosis and intracellular membrane trafficking [23–25]; the ERM proteins Ezrin, Radixin, and Moesin to cause cytoskeleton dissociation from the plasma membrane [26]; the intermediate filament protein vimentin [27]; and cyclophilin A [28]. Targeting of Ras is directly linked to cytotoxicity of host cells [29]. Varied cell types display different susceptibility [30] and substrate specificity [31,32]. Together, these activities of ExoS to contribute to barrier function loss, inhibition of phagocytosis, and cell death. The ADPr activity of ExoS can also auto-ADP ribosylate itself at residue R146 to inactivate its own RhoGAP activity [33].

Despite this information about ExoS and ExoT, many publications by us and others over several decades have demonstrated that *P*. *aeruginosa* strains encoding both exotoxins (invasive strains) can invade and thrive inside a variety of host cell types [34–37]. This includes multiple infection sites *in vivo*, such as the cornea, lung and bladder [36–38]. Yet, the intracellular lifestyle of *P*. *aeruginosa* is much less well understood than that of other facultative intracellular pathogens due to the persistent dogma that *P*. *aeruginosa* is an extracellular pathogen. This is probably related to the anti-phagocytic and cytotoxic potential of the T3SS effectors [13,19,20,39]. However, work by us and others has shown that the ADPr domain of ExoS is actually required for invasion and intracellular survival in both epithelial cells and neutrophils [40,41], and can even confer this capacity on some cytotoxic strains that do not naturally encode ExoS [42]. With respect to epithelial cells, the ExoS ADPr domain supports intracellular replication [40], inhibits lysosomal acidification that otherwise suppresses bacterial viability [43], counters autophagy [44], and induces the formation of low viscosity stable membrane blebs to which bacteria traffic [35]. These bacteria-containing blebs are distinct from apoptotic blebs, and can disconnect to form extracellular vesicles that continue to protect contained bacteria against membrane impermeable antibiotics [36].

Helping resolve this apparent contradiction about the functions of ExoS, we recently reported the bistability of T3SS expression results in only some bacteria expressing the T3SS/ExoS before internalization [42]. After the epithelial cell is invaded by *P*. *aeruginosa*, those entering the cytosol strongly express the T3SS as they rapidly divide [42], and disseminate throughout the host cell cytoplasm in a type 4 pili-dependent manner [45].

The aim of this study was to understand how the cytotoxic activities of the T3SS relate to its role in supporting a viable intracellular niche for *P*. *aeruginosa* after invasion occurs. Methods involved combined use of quantitative imaging and fluorescent probes to visualize and monitor bacterial location, T3SS expression, and host cell viability over time, with and without antibiotics varying in cell permeability to selectively study the impact of intracellular versus extracellular bacteria. The results unexpectedly showed that epithelial cells invaded by wild-type *P*. *aeruginosa* strain PAO1 remained viable longer than cells invaded by mutants lacking the ExoS, ExoT and ExoY T3SS effectors. Suppression of host cell death was due to ExoS ADPr activity, enabling time for intracellular replication. Induction of T3SS expression only after cell invasion recapitulated the ExoS-dependent intracellular phenotype, showing that intracellularly-expressed T3SS effectors were sufficient to drive intracellular pathogenesis. ExoS ADPr activity was also found to override the antiphagocytic activities of the RhoGAP domain in both ExoS and ExoT to allow cell invasion, providing more information about how the T3SS supports the intracellular lifestyle of *P*. *aeruginosa*. These ExoS-mediated phenotypes associated with intracellular *P*. *aeruginosa* differ from the host cell death shown to occur when ExoS is introduced by extracellular bacteria.

## Results

### Cells invaded by *P*. *aeruginosa* T3SS effector mutants become permeabilized more rapidly than cells invaded by wild-type bacteria

To broadly explore the relationship between cytotoxic activities of the T3SS effectors (ExoS, ExoT and ExoY) versus their roles in intracellular survival, wild-type invasive *P*. *aeruginosa* strain PAO1 was compared to an isogenic triple effector-null mutant PAO1Δ*exoSTY* for effects on epithelial cells over time. While lacking the three known T3SS effector toxins, the Δ*exoSTY* mutant still encodes the T3SS secretion machinery. After allowing bacteria to invade cells for 3 h, the non-cell permeable antibiotic amikacin was added to kill extracellular bacteria. Time-lapse imaging was then performed in the continued presence of amikacin up to 20 h post-infection to study events driven by the remaining (intracellular) bacteria. Intracellular T3SS-expressing bacteria were visualized using a T3SS-GFP reporter (previously validated for this purpose) and the timing of lytic cell death monitored using propidium iodide (PI) [42,46]. Host cell death was quantified for all cells in each field utilizing a customized Image J macro that tracks Hoechst labeling (all nuclei) and PI-labeling (nuclei of only permeabilized/dead) cells (see Image Analysis under Materials and Methods). Movies of nuclei staining experiments are shown (S1 Video).

**Fig 1:**
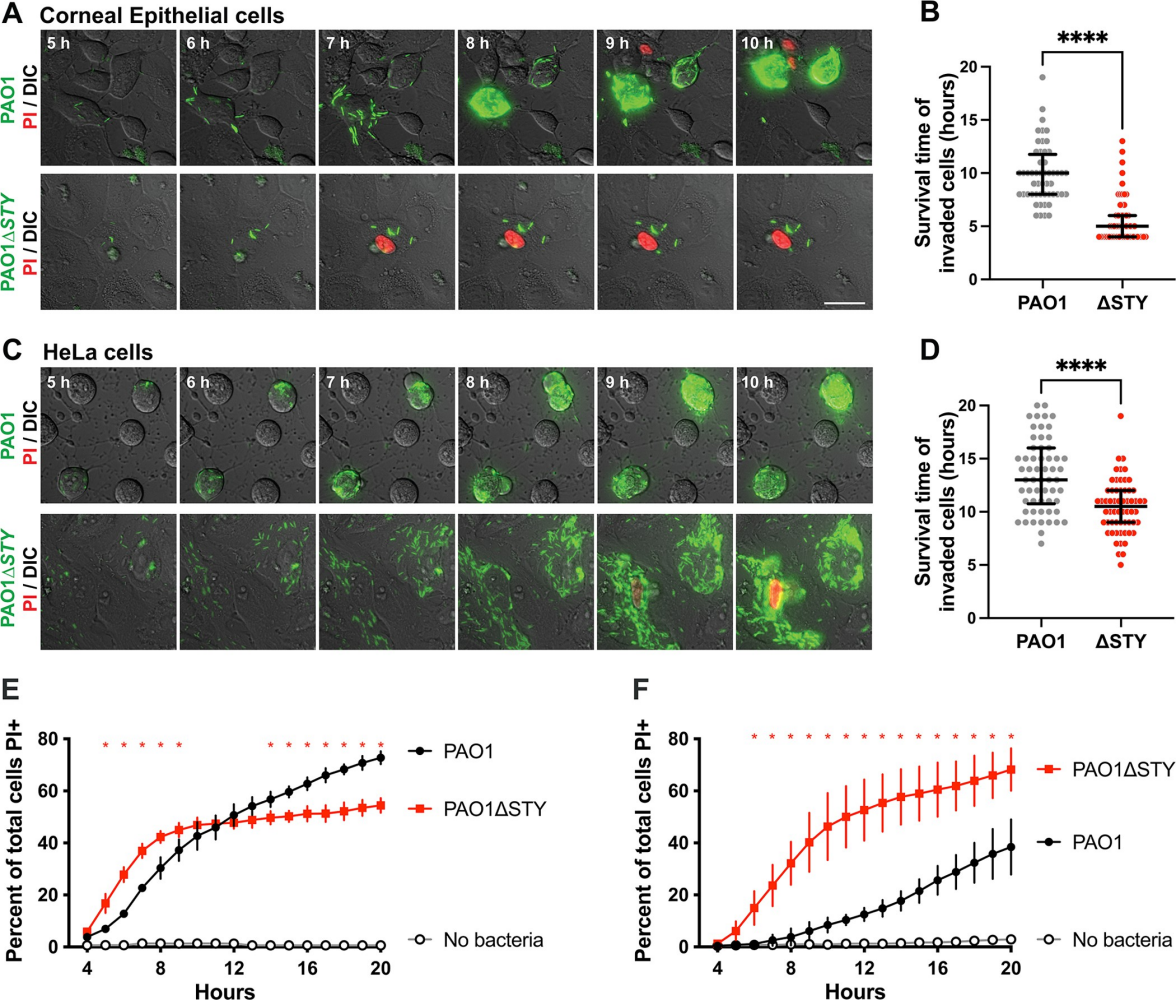
Host cell death limits bacterial intracellular replication. **(A)** Corneal epithelial cells were infected with PAO1 or PAO1Δ*exoSTY* expressing a T3SS-GFP reporter (pJNE05), then extracellular bacteria were eliminated with amikacin after 3 h. Propidium iodide (PI) was included in the media to detect cell membrane permeabilization. Time-lapse images were captured once per hour from 4 to 20 h post-infection. Scale bar = 25 μm. **(B)** Individual invaded cells were observed manually, and time of death determined. Data are expressed as the median with inter-quartile range (IQR), **** P < 0.001, Mann-Whitney U test. **(C-D)** HeLa cells were infected, imaged and analyzed as in panels A-B. **(E-F)** Corneal epithelial cells **(E)** or HeLa cells **(F)** were infected with wild-type or PAO1Δ*exoSTY* for 3 h and extracellular bacteria eliminated with amikacin. Hoechst and PI were included in the media and time-lapse images captured once per hour. Hoechst-positive and PI-positive nuclei were counted with a custom ImageJ macro at each time point and PAO1 compared to PAO1Δ*exoSTY*. Red asterisks above a given time point indicate P < 0.05, Student’s t-Test.

Previously, we reported that after wild-type PAO1 invades corneal epithelial cells it escapes from vacuoles to access the cytosol, where it replicates rapidly while strongly and consistently expressing the T3SS [35,40,42,43]. While doing so, pilus-dependent twitching motility promotes dissemination throughout the cytoplasm and flagella-mediated swimming allows for movement inside plasma membrane blebs [35,45]. The results confirmed this phenotype for wild-type PAO1 (Fig 1A), and invaded corneal epithelial cells did not label with PI for several hours (Fig 1). This showed that corneal epithelial cell membranes remained intact while containing T3SS-positive wild-type PAO1, during which time the number of individual cytoplasmic bacteria increased over time. Results with the mutant lacking all three effector toxins (PAO1Δ*exoSTY*) confirmed our published data in showing limited accumulation of observed bacterial cell numbers after corneal epithelial cell invasion (Fig 1A) [35,40]. Others have reported that T3SS-effector null mutants can be as, or even more, toxic to host cells as wild-type bacteria [47,48]. Here, we found the same result even when extracellular bacteria were inhibited with an antibiotic, with PAO1Δ*exoSTY* showing more frequent and earlier PI labeling than infection with wild-type PAO1. Together, these results showed that one or more of the T3SS effectors delays the death of cells infected with wild-type PAO1.

Movies of example fields from the experiments in Fig 1A are shown in S2 Video. Visual quantification of invaded host cell death up to 20 h post-infection confirmed that corneal epithelial cells containing intracellular PAO1 survived significantly longer than PAO1Δ*exoSTY*: median with inter-quartile range (IQR) time of cell death for PAO1Δ*exoSTY* was 5.2 ± 1.4 h which extended to 9.5 ± 1.3 h for PAO1 (Fig 1B). Whole population results confirmed less cell death for wild-type versus PAO1Δ*exoSTY* between 4 and 10 h (Fig 1E).

We previously reported that HeLa cells differ from corneal epithelial cells in supporting extensive intracellular replication by PAO1Δ*exoSTY* [42]. Tracking cell viability using PI labeling, here we found that successful replication by PAO1Δ*exoSTY* in the cytoplasm of HeLa cells (5–10 h post-infection), corresponded with less membrane permeabilization (Fig 1C and 1D). Nevertheless, there was still a difference between wild-type PAO1 and PAO1Δ*exoSTY* in rates of HeLa cell membrane permeabilization, albeit less striking than for corneal cells, with significantly longer survival over 20 h of observation post-infection for wild-type infected cells; mean (with SD) time of cell death was 10.2 ± 1.3 h for PAO1Δ*exoSTY* versus 13.0 ± 1.3 h for PAO1 (Fig 1D). Cell death across the population was lower for wild-type at all time points up to 20 h (Fig 1F). These results confirmed that one or more T3SS effector toxins encoded by PAO1 can counter the death of HeLa cells, in addition to corneal epithelial cells.

**Fig 2:**
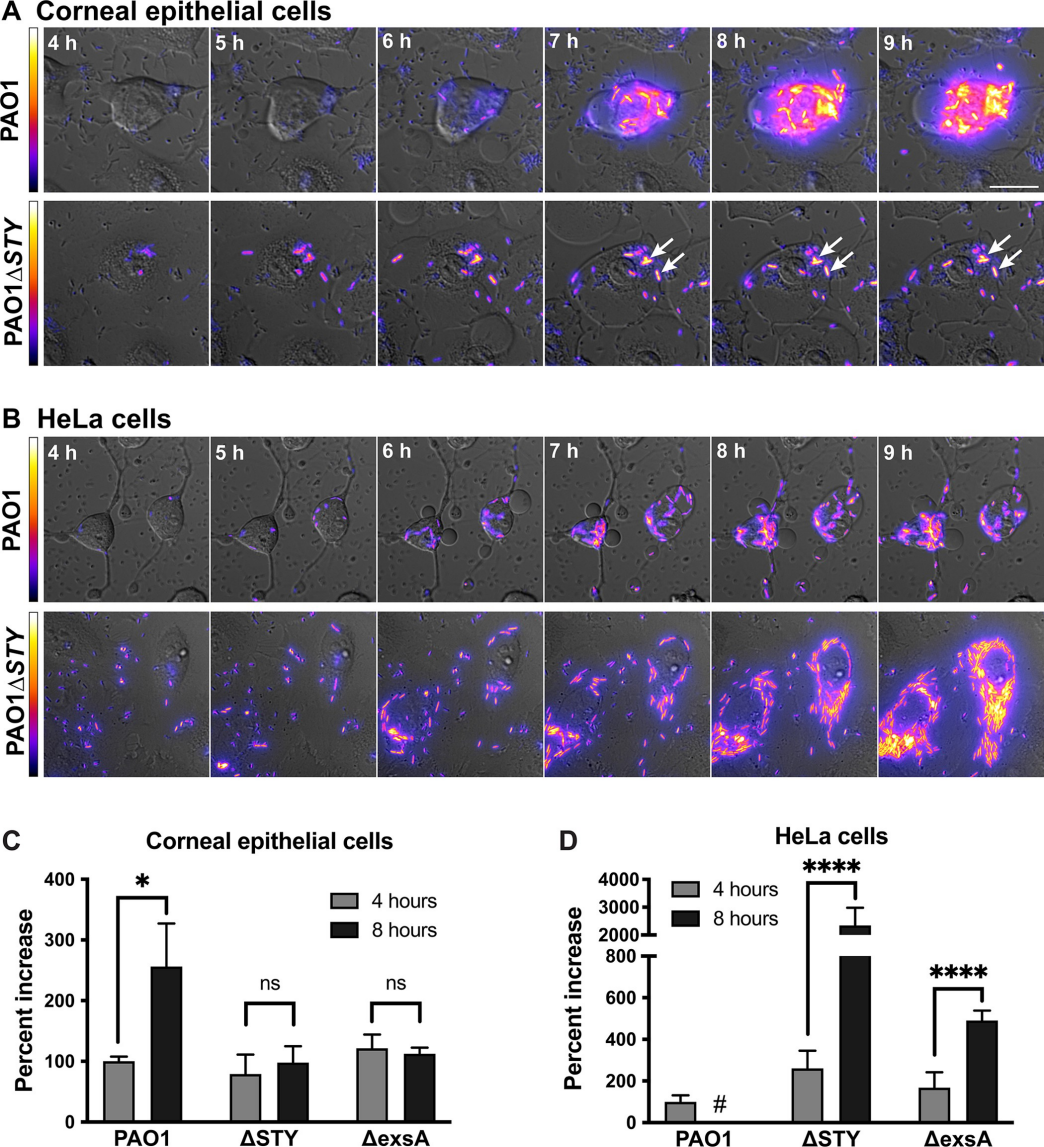
Corneal epithelial cells, but not HeLa cells, limit intracellular replication in the absence of type three effector toxins. Corneal epithelial cells **(**hTCEpi) **(A)** or HeLa **(B)** cells were infected with PAO1 or PAO1Δ*exoSTY* expressing the T3SS-GFP reporter (pJNE05) for 3 h, then extracellular bacteria eliminated with amikacin. Time-lapse images were captured hourly from 4 to 20 h post-infection. Scale bar = 25 μm. Arrows indicate stationary bacteria between captured time points. Heat-map shows T3SS-GFP reporter intensity of the bacterial cells. Corneal epithelial cells **(C)** or HeLa cells **(D)** were infected with PAO1, PAO1Δ*exoSTY* or PAO1Δ*exsA* (a T3SS negative control) for 3 h, then extracellular bacteria eliminated with gentamicin. Intracellular bacteria were collected from lysed cells at 4 or 8 h post-infection, and CFU counted by plating dilution series. Data expressed as mean ± SD, * P < 0.05, **** P < 0.001, ns = not significant, Student’s t-Test. # denotes not performed per cell fragility (see text and S1 Fig).

Knowing the differences in rates of host cell membrane permeabilization, we next quantified the ability of PAO1 and its effector mutants to replicate in HeLa and corneal epithelial cells. Intracellular replication was visualized using time-lapse imaging (Fig 2A and 2B) and antibiotic exclusion assays (Fig 2C and 2D). Fluorescent bacteria are displayed with a heat map lookup table in order to distinguish individual bacterial bodies. Results showed accumulation of wild-type PAO1 bacteria in both HeLa and corneal epithelial cells. However, PAO1Δ*exoSTY* was able to replicate in HeLa cells, but not corneal epithelial cells. This result corresponds with faster death of invaded corneal epithelial cells (Fig 1A and 1C), and would be consistent with membrane lysis allowing entry of extracellular antibiotic. Gentamicin protection data for wild-type PAO1 were not included in Fig 2D since examination of PAO1-infected HeLa cells showed significant dislodgement of cells by the washing steps necessary for antibiotic exclusion assays (S1 Fig), such that quantifying bacteria in remaining cells would have underestimated the number present prior to washing.

Closer inspection of these movies showed that for PAO1Δ*exoSTY*, PI staining occurred shortly after bacteria began cytoplasmic spread, which corresponded in time with loss of bacterial motility and replication. To visualize early events preceding cell death, time-lapse images were taken from 3 h to 6 h with imaging every 5 min for wild-type PAO1 and PAO1Δ*exoSTY* (S3 Video). Cell death appeared to occur after cytoplasmic entry and intracellular spreading for PAO1Δ*exoSTY*, whereas cells remained alive when invaded by wild-type PAO1. These results highlighted that continued bacterial survival inside epithelial cells in these assays depends on the host cell maintaining an intact membrane, otherwise they are killed by the antibiotic amikacin used to inhibit extracellular bacteria. Taken together, these results suggest that the lack of replication by T3SS effector-null mutants in corneal epithelial cells is a consequence of membrane permeabilization that allows antibiotic entry. The data further suggest that one or more of the effectors delays membrane permeabilization/death of both corneal and HeLa epithelial cells.

### Multiple effector enzymatic domains can counter host cell membrane permeabilization

**Fig 3:**
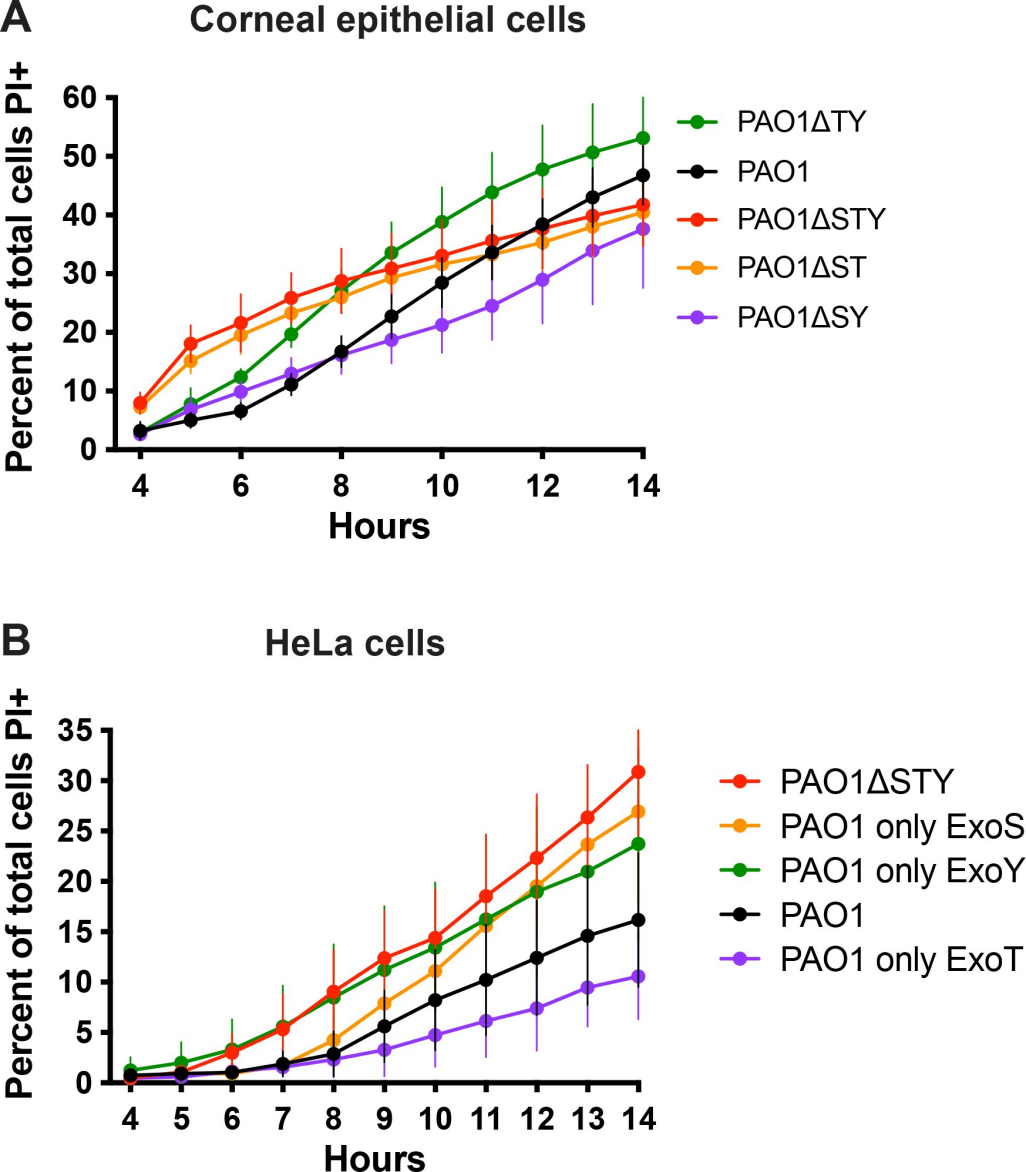
ExoT and ExoS improve survival time of infected cells over the effector-null mutant. Corneal epithelial cells (hTCEpi) **(A)** or HeLa cells **(B)** were infected with PAO1, PAO1Δ*exoSTY*, or mutants missing two of three known effectors for 3 h, then extracellular bacteria eliminated with amikacin. Hoechst and PI were included in the media. Time-lapse images were captured once per hour. Hoechst-positive and PI-positive nuclei were counted at each time point with an ImageJ macro. Data expressed as mean ± SD. Results shown are combined from at least three independent experiments.

To determine which of the three effectors suppress host cell permeabilization/death, corneal or HeLa epithelial cells were infected with a series of genomic double-knockout mutants each able to express only one effector. Fig 3A shows results for corneal cells. Compared to PAO1*ΔexoSTY* lacking all three effectors (red line), bacteria expressing only ExoT (PAO1Δ*exoSY*, purple line) cause less death of corneal cells up to 14 h post-infection. Bacteria expressing only ExoS (PAO1Δe*xoTY*, green line) also caused less cell death from 4–7 h post-infection and then more cell death after 9 h (green line). A similar pattern was observed in HeLa cells (Fig 3B) although ExoS delayed cell death for a longer period (up to ~ 10 h post-infection) and did not surpass PAO1*ΔexoSTY* at later time points. In both cell types, ExoT alone caused even less cell death than wild-type PAO1 from 8 h onwards, while ExoY alone (PAO1Δ*exoST*) had little effect on cell death rate (Fig 3A and 3B, orange line). Thus, either ExoS or ExoT was sufficient to reduce the rate of host cell death.

**Fig 4:**
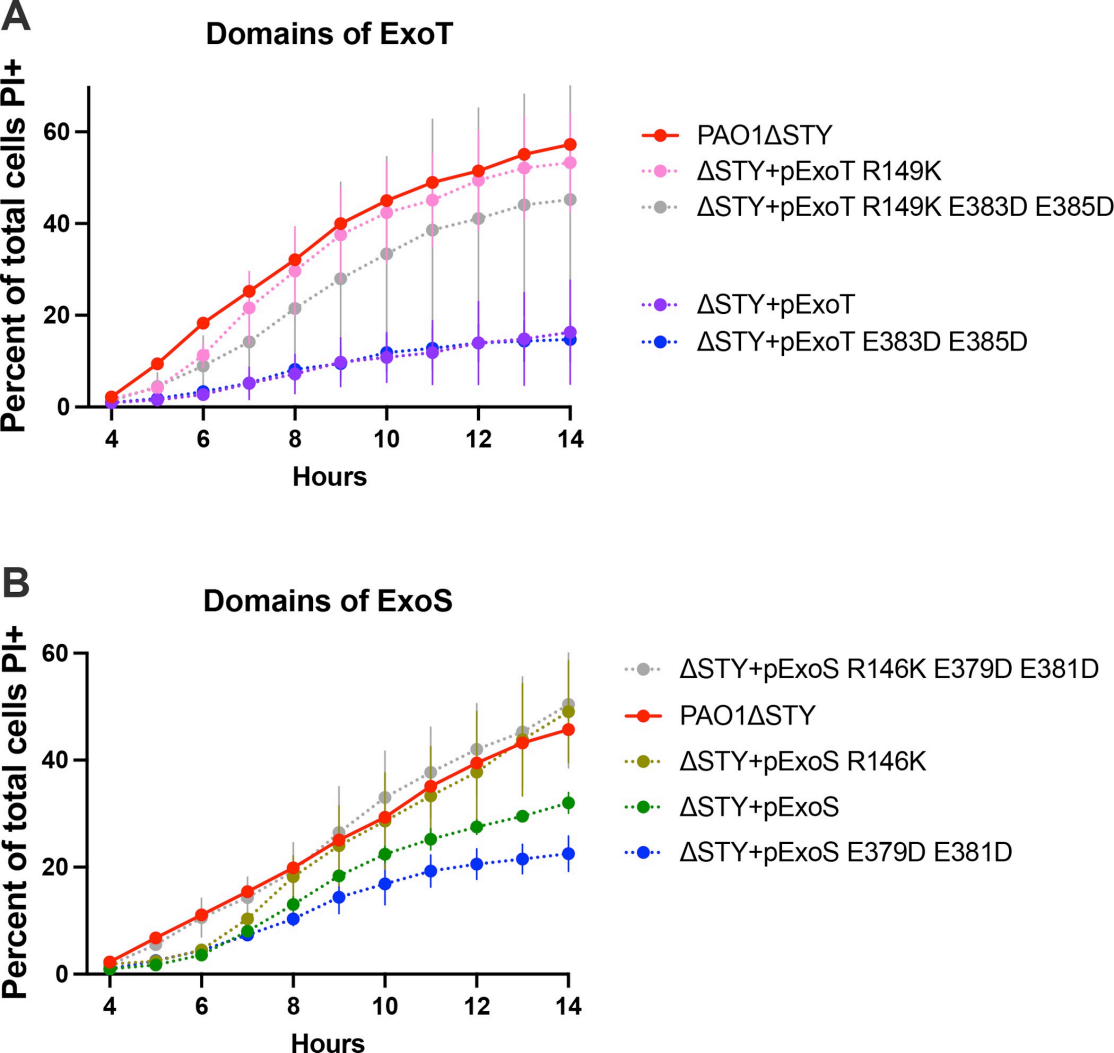
RhoGAP domains of ExoT or ExoS and the ExoS ADPR domain reduce or delay host cell death. HeLa cells were infected with PAO1Δ*exoSTY* complemented with indicated plasmids to determine the role of individual domains of ExoT **(A)** or ExoS **(B)** for 3 h, then extracellular bacteria eliminated with amikacin. Hoechst and PI were included in the media. Time-lapse images were captured once per hour beginning at 4 h. Hoechst-positive and PI-positive nuclei were counted with ImageJ macro at each time point. Data are expressed as the mean ± SD. Results shown are combined from at least three independent experiments. Note: SD from PAO1Δ*exoSTY* (red line) was removed for better visualization of variability among domain mutants.

Since ExoS and ExoT have dual enzymatic activities (RhoGAP and ADPr), we next explored which were involved. ExoT, with point mutations that inactivate either the RhoGAP activity (R149K) or ADPr activity (E383D/E385D), or both, was expressed in PAO1Δ*exoSTY* from the pUCP18 vector [21,22]. HeLa cells were used, given the more consistent separation between wild-type PAO1 and effector mutant phenotypes described above. Results showed that complementation of PAO1Δ*exoSTY* with ExoT intact, or with only RhoGAP activity, greatly limited the rate of host cell death from 4–12 h post infection (Fig 4A). In contrast, catalytic-null ExoT, or ExoT with only ADPr activity, had no impact compared to PAO1Δ*exoSTY*. To study ExoS, PAO1Δ*exoSTY* expressing ExoS lacking RhoGAP activity (R146K), ADPr activity (E379D/E381D), or both were used (Fig 4B) [49–51]. Similar to ExoT results, fully functional ExoS or only the RhoGAP domain of ExoS was sufficient for limiting the rate of cell death over 14 h. Differing from ExoT, the ExoS ADPr activity was also sufficient to delay cell death (from 4–7 h post-infection): not observed after complementation with catalytic-null ExoS (Fig 4B). Thus, the RhoGAP activity of either ExoS or ExoT was sufficient to limit host cell death after *P*. *aeruginosa* exposure, while the ADPr activity of ExoS delayed host cell death occurring at later timepoints.

### ExoS ADPr activity delays cell death to promote intracellular replication

**Fig 5:**
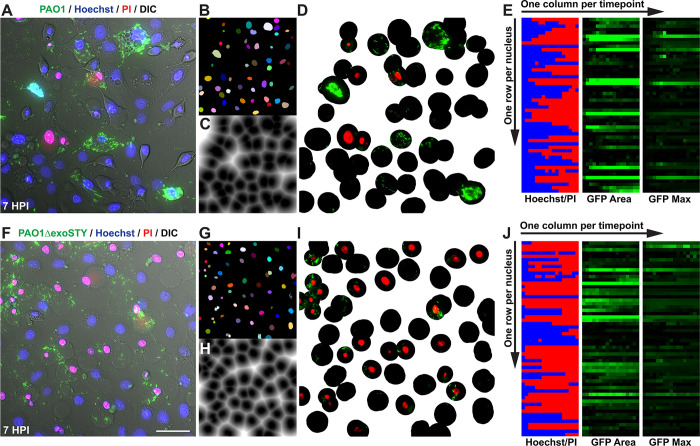
Time-lapse imaging computational analysis. For each time-lapse field for PAO1 **(A)** versus PAO1Δe*xoSTY* **(F)**, individual nuclei were segmented into ultimate points, tracked by mTrack2 plugin, and assigned an ID number **(B)** versus **(G)** respectively. Nuclei were excluded if tracks were not present in all frames. A distance map was drawn from each nucleus to designate its cytoplasmic area **(C)** versus **(H)**. A distance limit of 50 was determined empirically based on average corneal epithelial cell size **(D)** versus **(I)**. Some bacteria were excluded from analysis when located further from the nucleus but prevented from being assigned to neighboring cells using this limit. Measurements were recorded as a series of TIF images with one row per nucleus, and one column per timepoint **(E)** versus **(J)**. The ratio of PI to Hoechst signal was measured for each nucleus. GFP area, max intensity, and median intensity were measured within each cell’s defined area. A value to indicate cell death was determined empirically based on value changes when PI became visible in corresponding images, and background values for GFP area and GFP max were determined using uninfected controls and fields where bacteria were not present.

We and others have shown that the T3SS needle and T3SS effectors can elicit cytotoxic effects when delivered by extracellular bacteria, potentially complicating experiments designed to understand host cell death in response to bacterial invasion. To correlate the timing of host cell death (Figs 3 and 4) specifically with intracellular presence and persistence of bacteria (Figs 1 and 2) a computational image analysis method was developed to complement the manual method used in Fig 1. This allowed automated individual cell analysis, and enabled objective comparison of more conditions and greater numbers of imaged fields than can be done manually. Specifically, this method tracked individual nuclei, reported the ratio of Hoechst-positive nuclei to PI in each frame to determine the timepoint of death, and simultaneously reported the presence of GFP-positive bacteria appearing the in the host cell cytoplasm proximal to the nucleus (Fig 5, see Image Analysis under Materials and Methods for further details).

**Fig 6:**
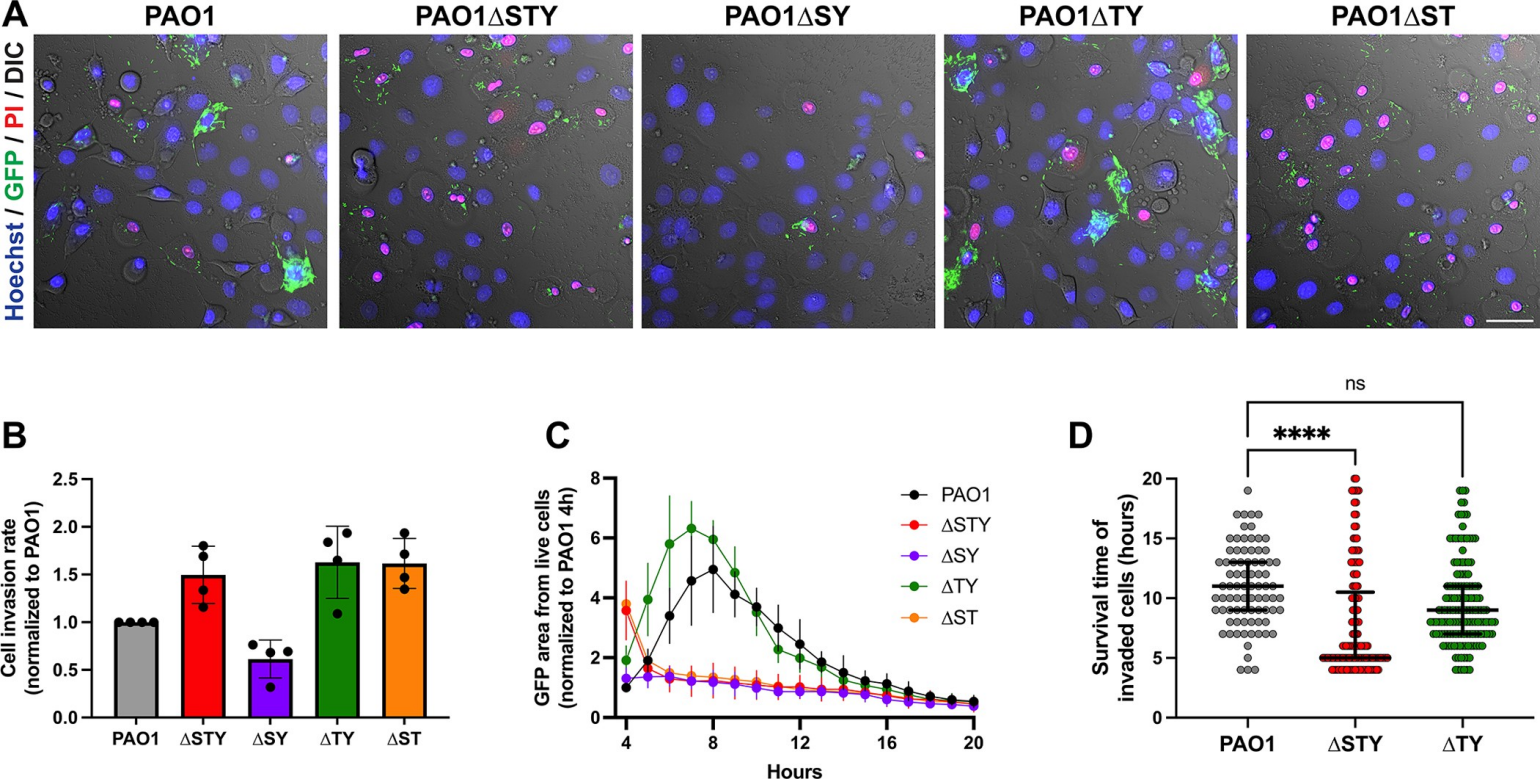
ExoS-mediated intracellular replication coincides with delayed host cell death. **(A)** Corneal epithelial cells were infected with PAO1, PAO1Δ*exoSTY*, or mutants missing two effectors for 3 h, and extracellular bacteria were eliminated with amikacin. Hoechst and PI were included in the media and bacteria expressed GFP under the T3SS-GFP reporter pJNE05. Time-lapse images were captured once per hour. Representative images from 7 h post-infection are shown. **(B)** Individual invaded cells were identified through a FIJI analysis macro (Fig 5) in four experimental replicates, and total number of invaded cells normalized to PAO1 to show how each effector altered internalization when expressed in isolation. As expected, fewer invaded cells were observed if only ExoT was expressed. **(C)** The area of GFP signal contained within only cells designated as “alive” at each time-point was summed and expressed as a mean ± SD of four replicates over 20 h. **(D)** The time-point at which nuclei of invaded cells became PI+ was determined. A representative experiment of four is shown. See also S4 Video. Data expressed as median with IQR, ** P < 0.01, **** P < 0.001, ns = not significant, Kruskal-Wallis test.

Corneal epithelial cells were infected with wild-type PAO1, PAO1Δ*exoSTY*, or mutants expressing individual T3SS effectors (as in Fig 3). Only corneal cells were used here because of the greater difference in survival time after invasion for wild-type versus triple effector mutants (Fig 1B) and bigger impact of T3SS effectors on intracellular replication (Fig 2C). Experiments were performed as above. Time-lapse still images from 7 h post infection (Fig 6A) showed that bacteria expressing only ExoS (PAO1Δ*exoTY*) were similar to wild-type PAO1 in intracellular localization within viable host cells (no PI labeling). In contrast, few bacteria became intracellular when expressing only ExoT (PAO1Δ*exoSY*), corresponding with less host cell death compared to either PAO1Δ*exoSTY* or PAO1Δ*exoST*. These phenotypes are shown in S4 Video. Comparison of the total number of invaded cells between four replicates (Fig 6B) confirmed that ExoT in isolation reduced invasion below wild-type PAO1 consistent with its known anti-internalization activity [19,20]. Mutants lacking ExoT invaded more cells.

**Fig 7:**
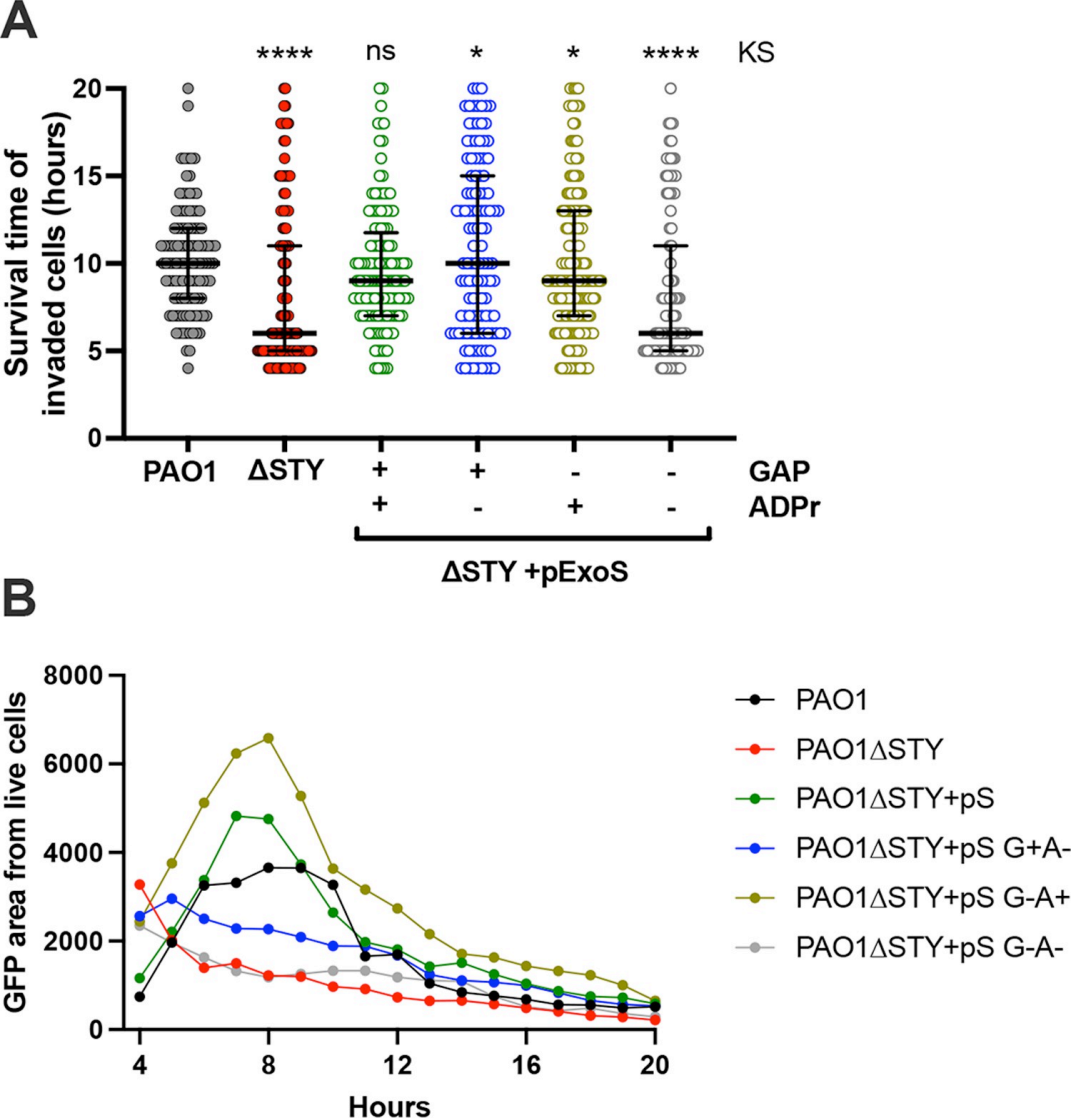
ExoS ADPr domain prolongs invaded host cell life and is sufficient for niche utilization. **(A)** Corneal epithelial cells were infected with PAO1, PAO1Δ*exoSTY* or PAO1Δ*exoSTY* complemented with ExoS on a plasmid with indicating enzymatic activities for 3 h, and extracellular bacteria then eliminated with amikacin. Hoechst and PI were included in the media and bacteria expressed the T3SS-GFP reporter. Time-lapse images were captured hourly from 4 to 20 h. Individual invaded cells were identified through the FIJI analysis macro and the time when nuclei became PI+ determined. Experiments were repeated twice, and a representative experiment is shown. **(B)** The area of GFP signal contained within only cells designated as alive was summed and plotted from the same data set as panel (A). Complementation of PAO1Δ*exoSTY* with ExoS or ExoS with an active ADPr domain was associated with a significant intracellular presence similar to PAO1. Host cell survival data expressed as the median with IQR. Kolmogorov-Smirnov (KS) significance values versus PAO1, * P < 0.05, ** P < 0.01, **** P < 0.001.

To further quantify intracellular bacteria in relation to host cell viability, the accumulation of GFP pixel area within each designated cytoplasmic boundary (reflecting the quantity of GFP-expressing intracellular bacteria) was summed in each experimental replicate from cells designated as “alive” by the absence of PI signal (Fig 6C). Cells containing intracellular wild-type PAO1 (Fig 6C, black line) or PAO1Δ*exoTY* mutants (i.e. expressing only ExoS) (Fig 6C, green line) showed increasing GFP area up to 7 to 8 h post-infection. GFP area did not increase over time for other mutants (all lacking ExoS), despite a greater initial GFP area (at 4 h post-infection) for triple effector mutants and those expressing only ExoY (Fig 6C, red line and orange lines); the latter presumably due to a higher percentage of invaded cells, initially. The small GFP area for mutants expressing only ExoT (Fig 6C, purple line) aligned with its previously discussed anti-internalization activity.

Next, the survival time of cells containing intracellular bacteria was determined. Bacteria that only express ExoS (PAO1Δ*exoTY*) were compared to wild-type PAO1 and the triple effector-null mutant (Fig 6D). Expression of ExoS alone was associated with a significant increase in survival time versus the effector-null mutant, with no significant difference between bacteria expressing only ExoS and wild-type.

Together, the data shown in Fig 6 suggest that the primary mechanism by which ExoT alone protects host cells against permeabilization involves keeping the bacteria extracellular. In contrast, ExoS-mediated intracellular replication correlates with an ExoS-mediated delay in host cell death, the latter driven by intracellular bacteria in the absence of known T3SS effectors.

Since both catalytic domains of ExoS were able to delay cell death (as shown above), additional experiments were performed to study them separately, using complementation in a PAO1Δe*xoSTY* background (Fig 7A and 7B). As expected, complementation with intact ExoS restored host cell survival time to that of wild-type PAO1 (Fig 7A), and an ExoS mutant lacking ADPr and RhoGAP activity mirrored the effector-null mutant. Interestingly, mutation of either one of the two domains increased host cell survival time (Fig 7A). However, when the analysis was done using intracellular GFP area (internalized bacteria, invaded cells), only ADPr-positive ExoS (i.e. intact ExoS or RhoGAP mutant ExoS) matched the wild-type PAO1 phenotype (up to 8 h post-infection). These results showed that while the ADPr activity of ExoS could protect invaded cells against cell death, protection provided by its RhoGAP domain (apparent only in the absence of its ADPr domain) was attributable to its antiphagocytic activity, similar to the RhoGAP domain of ExoT.

### Modulation of *P*. *aeruginosa* invasion by the ExoS RhoGAP domain is limited by ExoS auto-ADPr activity

Fig 6 experiments studied the relationship between internalization and cell death for isolated effector toxins; thus, in Fig 7 we asked which enzymatic domains of ExoS aid in intracellular persistence after invasion. In addition to showing that ADPr activity of ExoS delays host cell death that otherwise follows *P*. *aeruginosa* entry into the cytosol in corneal cells, the results also suggested that ExoS ADPr activity can override the antiphagocytic activities of ExoS and ExoT RhoGAP to promote bacterial invasion.

**Fig 8:**
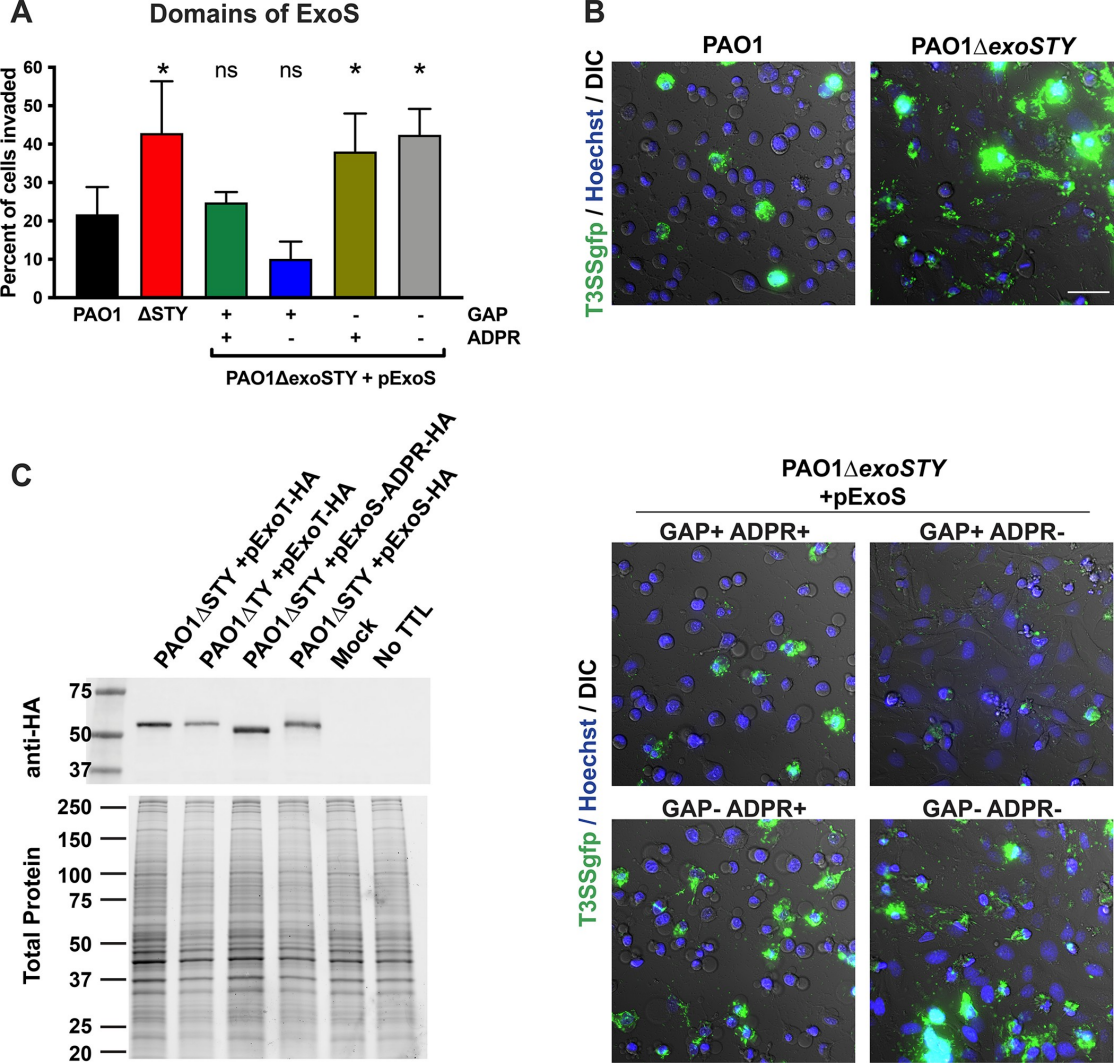
Modulation of *P*. *aeruginosa* invasion by the ExoS RhoGAP domain is limited by ExoS auto-ADPr activity. **(A)** HeLa cells were infected with indicated strain and mutants for 3 h and extracellular bacteria eliminated with amikacin. Bacteria expressed GFP from the T3SS GFP-reporter plasmid pJNE05 and time-lapse images used to determine the percent of cells invaded. Data expressed as mean ± SD. * P < 0.05, ns = not significant. One-way ANOVA. **(B)** Examples of time-lapse frames at 10 h shows relative quantity of invaded cells in an example field. Scale bar = 50 μm. **(C)** Corneal epithelial cells were infected with indicated strain and plasmid, and either HA-tagged ExoT or ExoS detected by Western blot at 4 h post-infection. Biotin-NAD was pulsed into the cells by transient tetanolysin (TTL) permeabilization to enhance detectable size shift that was evident with ExoS, but not ExoT. A representative experiment of two is shown.

Studying the impact of ExoS and ExoT on *P*. *aeruginosa* invasion has involved use of standard antibiotic exclusion assays that quantify total number of intracellular bacteria in a population of infected cells [19,20,40,42]. That total is a function of both number of invaded cells and replication rate within them, which can be differentially impacted by T3SS effector activities as shown above. Here, we continued our use of imaging to determine how effector enzymatic activities influence the percentage of cells invaded. Infected HeLa cells were treated with amikacin to kill extracellular bacteria followed by imaging of the intracellular bacteria (GFP-expressing) to determine the percentage of cells that had been invaded [42]. PAO1Δ*exoSTY* invaded a greater percentage of cells than wild-type PAO1 (Fig 8A and 8B). Plasmid complementation of PAO1Δ*exoSTY* with ExoS showed that RhoGAP activity reduced the percentage of cells invaded from ~ 40% to ~ 10%, aligning with RhoGAP antiphagocytic activity. This level was below that of wild-type. The ExoS ADPr domain appeared to temper the antiphagocytic impact of the RhoGAP domain causing a greater percentage of cells to be infected and increasing the total number of bacteria invading as previously shown (Fig 8A and 8B).

ExoS auto-ADP ribosylates its own RhoGAP domain at Arg146, a required active site residue [33]. This post-translational modification can be detected by size shift. We examined whether this may occur in our present study by detecting HA-tagged ExoS in a background where ADPr activity of ExoS is absent (both genomically and by plasmid complementation) (Fig 8C, lane 3), compared to wild-type ExoS expressed by plasmid (Fig 8C, lane 4), where it can ADP-ribosylate itself *in trans*. The size shift observed is consistent with ExoS auto-ADP-ribosylation. This likely explains how the ADPr activity of ExoS overrides the anti-internalization activity of its own RhoGAP domain in earlier experiments [33]. With respect to how ExoS overrides the anti-phagocytic RhoGAP activity of ExoT, ADP-ribosylation by ExoS has also been hypothesized to occur [18]. We examined that possibility but found no discernable size shift of ExoT (Fig 8C, lanes 1 and 2). Thus, ExoT does not appear to be a detectable substrate of ExoS, at least in this *in vitro* study.

### Intracellular type three secretion is sufficient to enable an intracellular niche

**Fig 9:**
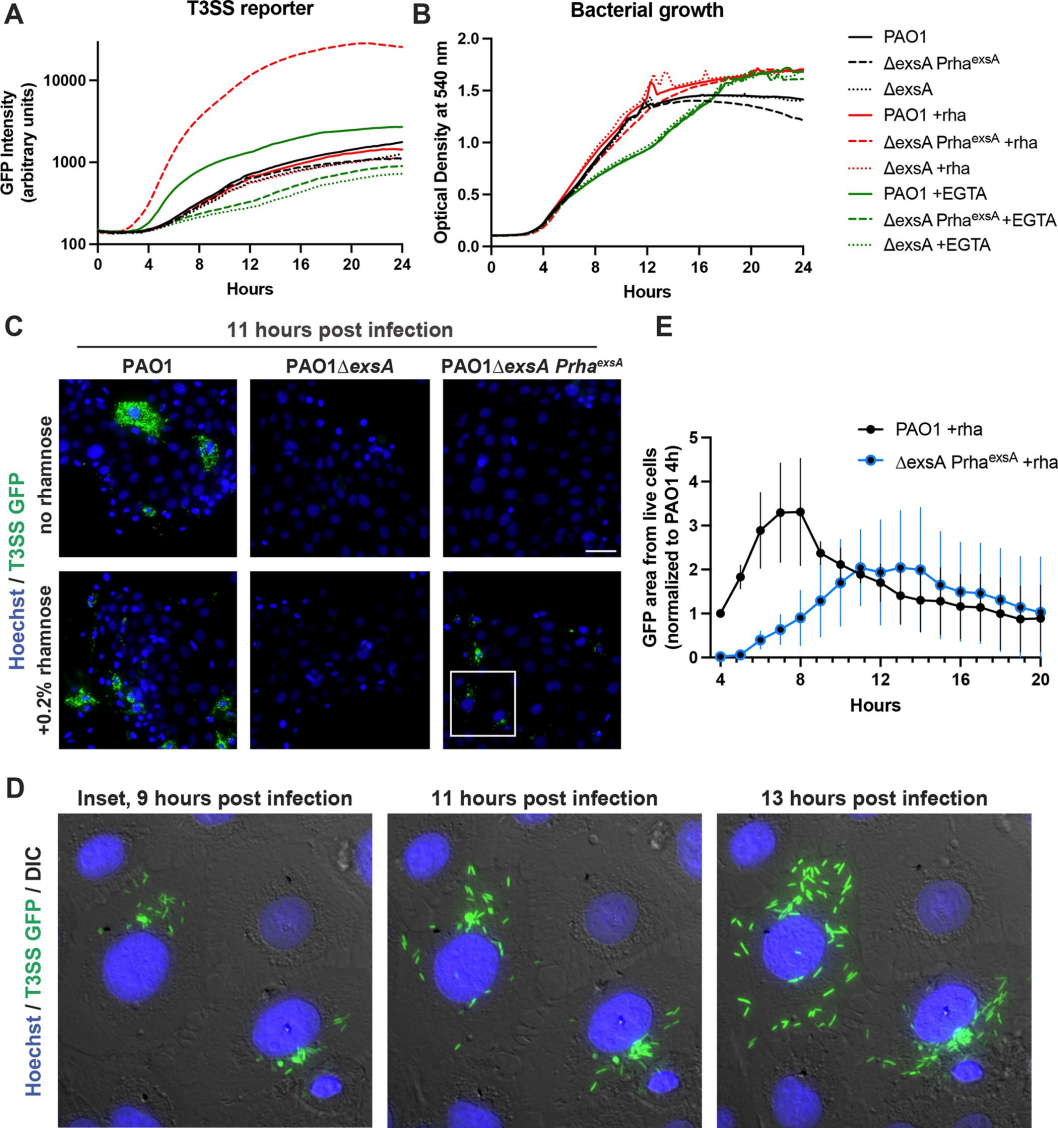
Intracellular induction of T3SS effector expression is sufficient to generate an intracellular niche. **(A)** A rhamnose-inducible ExsA construct was integrated at the Tn7 chromosomal site of PAO1Δ*exsA*. Strains were transformed with the T3SS-GFP reporter pJNE05. Strains were grown in TSB, TSB with 2 mM EGTA, or 0.2% rhamnose. GFP intensity measured at 15 min intervals showed effective T3SS induction. **(B)** Absorbance at 540 nm was measured simultaneously with the experiment in (A) to indicate bacterial growth. **(C)** Indicated strains were visualized in a time-lapse format and 11 h post-infection shown with Hoechst and GFP. **(D)** Insets of the same field of induced ExsA expression at 9 h, 11 h and 13 h post-infection show the accumulation of spreading intracellular bacteria. **(E)** The GFP area accumulation in only live cells was summed and normalized to PAO1 at 4 h. Three replicates are shown with mean ± SD.

Our results suggest that the ADPr activity of ExoS promotes host cell survival to the benefit of intracellular bacteria. To explore if intracellular expression of the T3SS is solely sufficient to form the replicative niches dependent on ExoS ADPr activity, we engineered a rhamnose-inducible ExsA expression construct (pUC18 Tn7 Prha^exsA^), which integrates into the *P*. *aeruginosa* chromosome at the Tn7 site. To verify this system, an *in vitro* growth assay was performed where T3SS activity was induced with either 2mM EGTA [46] or with 0.2% rhamnose. T3SS activity was measured by GFP fluorescence using the reporter pJNE05, and optical density of bacteria during the growth curves was measured at 540 nm. Rhamnose-dependent induction of the T3SS reporter verified ExsA expression from the Tn7 site in Δ*exsA* background (Fig 9A and 9B).

Bacteria were incubated with corneal epithelial cells for 3 h, extracellular bacteria eliminated with amikacin (for 30 min), and cells were then treated with 0.2% rhamnose to trigger expression of the T3SS in viable intracellular bacteria. Live imaging beginning at 4 h post-infection showed that rhamnose-induced intracellular PAO1Δ*exsA* Prha^exsA^ had become T3SS-positive and exhibited intracellular replication and spreading, mimicking wild-type PAO1 (Fig 9C and 9D), with maximum intracellular spreading occurring 2–3 h later than wild-type. Example movies are shown in S5 Video. GFP intensity from live cells was quantified (Fig 9E). While this does not entirely rule out involvement of extracellular bacteria, it does show that extracellular expression of the T3SS is not required for *P*. *aeruginosa* to construct a replicative niche inside epithelial cells.

**Fig 10:**
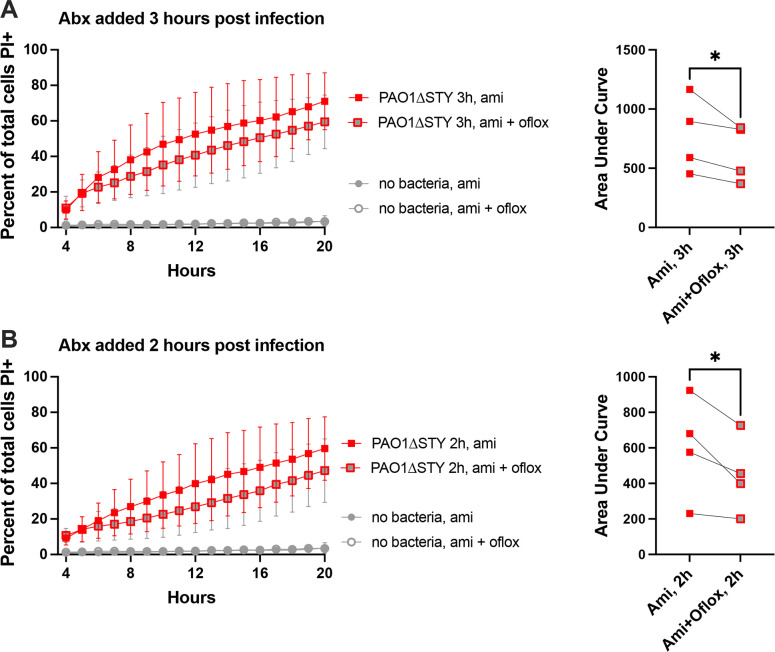
Ofloxacin treatment reduces host cell death after bacterial invasion. Corneal epithelial cells were infected with PAO1Δ*exoSTY* for (**A**) 3 h or (**B**) 2 h, and either 200 μg/mL amikacin alone, or 200 μg/mL amikacin combined with 25 μg/mL ofloxacin, added to eliminate extracellular bacteria only, or both extracellular and intracellular bacteria, respectively. Cell death was tracked using Hoechst and propidium iodide staining in time-lapse images from 4–20 h post-infection and plotted as a percentage. The addition of ofloxacin to kill intracellular bacteria reduced overall cell death. Four replicates are shown for each condition with area under the curve plotted for each replicate to demonstrate a consistent reduction in cell death with ofloxacin treatment. * P < 0.05 (paired Student’s t-Test).

Having shown that intracellular T3SS expression can enable intracellular persistence, we next asked if intracellular bacteria also contribute to driving/triggering the cell death that ExoS ADPr activity counters. This possibility is supported by ExoT RhoGAP activity inhibiting cell death and invasion, and also by the timing of cell death following bacterial entry into the cytosol. To directly explore whether viable intracellular bacteria drive cell death, cells were infected with PAO1Δ*exoSTY*. Both extracellular bacteria and intracellular bacteria were killed, using an additional cell permeable antibiotic ofloxacin, to determine if this would reduce subsequent host cell death. Results from four experimental replicates under two different conditions, i.e. addition of antibiotics at 3 h or 2 h post-infection, all showed that inclusion of ofloxacin to kill intracellular bacteria consistently reduced the rate of host cell death (Fig 10).

## Discussion

While *P*. *aeruginosa* is sometimes considered an extracellular pathogen, it can establish a niche inside a variety of mammalian cells [35,36,52,53]. For epithelial cells, this can depend on ExoS ADPr activity, which inhibits vacuole acidification, enables vacuolar escape, interferes with autophagy, and is required for the formation of membrane blebs niches and intracellular survival and replication [40,43,44]. Factors contributing to skepticism about *P*. *aeruginosa* replicating inside cells include the demonstrated antiphagocytic activity of its T3SS effectors, the unknown relationship between T3SS-mediated cytotoxicity and bacterial survival inside the cell, and that antibiotic-recalcitrant extracellular bacterial biofilm formation that could confuse results of antibiotic exclusion assays used to quantify intracellular bacteria.

Here, we used quantitative imaging to address these concerns. The results showed that epithelial cells can potentially limit intracellular replication of *P*. *aeruginosa* through lytic cell death when bacteria enter the cytosol. However, the data also showed that the ADPr activity of ExoS encoded by invasive *P*. *aeruginosa* strains can establish an intracellular niche to delay this cell lysis, thereby allowing many rounds of replication in the cytosol before the cell succumbs. This adds to the growing list of roles played by ExoS ADPr activity in supporting the intracellular lifestyle of *P*. *aeruginosa*. The ADPr domain of ExoT, which has different targets, was found to have no impact on the timing of cell lysis.

While the RhoGAP activity domain of both ExoS and ExoT also countered host cell death when expressed alone, this was associated with their capacity to inhibit bacterial internalization. Explaining how *P*. *aeruginosa* invades cells while expressing these two effectors with anti-phagocytic potential, the data showed that ExoS ADPr activity can override their capacity to block internalization. For ExoS, this was associated with auto-ADP-ribosylation of its RhoGAP domain. How ExoT RhoGAP anti-internalization activity is countered by the ADPr domain of ExoS does not appear to involve a similar post-translational modification, as predicted by the lack of a size shift. Possibilities include that secretion of multiple effectors serves as a rate-limiting step of ExoT-dominated effects, and/or ExoS inhibition of contact-dependent T3SS activation when bacteria encounter a previously-intoxicated cell [54].

In a previous publication, we attributed lack of replication by a T3SS effector mutant in corneal cells to their entrapment inside vacuoles, which appeared to be the case using phase contrast imaging [35]. Here, fluorescence imaging showed that they do access the cytoplasm and spread throughout the cell in small numbers. This was followed closely by PI staining of the nucleus, corresponding in time with a loss of bacterial motility and replication, reflecting entry of both PI and the antibiotic as a result of membrane permeabilization. In other words, the lack of T3SS effector-null mutant replication in corneal epithelial cells is a consequence of cell lysis allowing antibiotic entry, not necessarily an inability to exit vacuoles or survive in the cytoplasm. For wild-type bacteria expressing ExoS ADPr activity, delayed host cell permeabilization provides an opportunity to replicate in the cytosol for several additional hours.

In this study, we used an antibiotic whilst exploring the relationship between cell integrity and intracellular *P*. *aeruginosa*. Rather than being artifactual, this likely mirrors conditions encountered during corneal infection, with intracellular bacteria protected from antimicrobial peptides, complement, immune cells, antibodies, and sometimes also non-cell permeable antibiotics during treatment. At the ocular surface, bacteria residing within epithelial cells are protected from tear fluid and its antimicrobial properties [55], and from being swept away by blinking action.

We also asked if T3SS expression by intracellular bacteria was sufficient to support intracellular niche formation, supported by our data showing that intracellular *P*. *aeruginosa* strongly express the T3SS while they replicate in the cytosol [42]. The results showed that inducing T3SS expression only after bacteria were intracellular (using rhamnose induction of ExsA in a Δ*exsA* background), after extracellular bacteria had been killed, could rescue the entire phenotype of intracellular replication, confirming that T3SS expression by extracellular bacteria was not required to support the intracellular lifestyle of *P*. *aeruginosa* encoding ExoS.

While corneal epithelial cells died when T3SS effector mutants entered their cytosol, HeLa cells died more slowly, allowing several rounds of intracellular replication as we have previously reported [42]. This suggests a host cell contribution to permeabilization and death, with relevant differences between these two cell types. HeLa cells have known defects in pattern recognition, including the absence or lack of function of TLR4 and TLR5, and abnormalities in downstream responses [56,57]. Subsequent studies comparing corneal and HeLa cells might help decipher details of how epithelial cells respond to *P*. *aeruginosa*.

Rapid triggering of cell death occurred in the absence of ExoS, ExoT and ExoY, excluding their involvement in cytotoxicity. It is possible that other T3SS components are involved, as T3SS secretion components PscF and PscI can be recognized by mammalian cells to drive programmed cell death responses, e.g. inflammasome activation and associated pyroptosis which results in membrane permeabilization [58,59]. Since the receptors that recognize these components are located in cytosol of some cell types (e.g. human NAIP-NLRC4 inflammasome), their involvement would explain why intracellular bacteria contribute to cell death, and why the timing relates to cytosol entry. It is also possible that the T3SS plays no direct role in driving cell death, its only role being indirect in vacuolar escape/cytosol entry as we and others have shown [35,42,60]. If cell death is primarily driven by the host cell (as suggested by the HeLa cell results), other bacterial-associated triggers could include LPS, flagella, peptidoglycan or other surface associated factors recognized by cytosol receptors involved in innate immunity. For example, NOD1 and NOD2 respond to peptidoglycan components to activate NF-κB signaling leading to pro-inflammatory events and cytotoxic effects on host cells [61,62]. In addition to type III secretion system needle proteins, cytosolic human NAIP can recognize flagellin to cause NLRC4 inflammasome activation and pyroptosis [63]. Intracellular LPS can activate the caspase-4 inflammasome, at least in other cell types [64]. Since regulated cell death pathways are currently less-well characterized in epithelial cells than in innate immune cells, more research will be required before determining their relevance to this study.

With respect to the mechanism by which ExoS delays cell death, it might relate to its ability to inhibit IL-1β secretion, which is a marker of inflammasome activation [65]. However, efforts to determine how ExoS ADPr activity delays cell death will first need to focus on understand the underlying cell death itself, which may be complex for reasons discussed above. Also, challenging will be efforts to determine the substrates that ExoS acts up to accomplish this, given that its ADPr activity has broad substrate specificity [18,27].

While we have found that ExoS ADPr activity can protect against host cell lysis, ExoS is commonly considered a cytotoxin with activity against a variety of mammalian cells [51,66,67]. Of possible relevance, studies of ExoS cytotoxic activities have involved ExoS transfection (without bacteria) or extracellular delivery using *P*. *aeruginosa* strain PA103. PA103 is a cytotoxic strain that does not naturally encode ExoS. It does not invade cells when engineered to express ExoS (an unusual trait even for a cytotoxic strain), and therefore introduces ExoS into host cells from an extracellular location [42]. As a result, previous studies of ExoS effects on mammalian cells have generally been done without intracellular bacteria present. Here, we allowed *P*. *aeruginosa* to become intracellular, as occurs when ExoS is naturally encoded. Thus, seemingly contradictory effect of ExoS ADPr activity might depend on the circumstances, e.g. the location of the bacteria when ExoS is secreted.

*P*. *aeruginosa* encodes a complex array of regulatory systems that allows it to adapt to a wide range of environmental conditions, to adhere to almost any surface, to excel at forming biofilms, and inherently resist killing. These properties make it a difficult pathogen to work with in *in vitro* assays, making it fraught with opportunity for discovering artifacts, misinterpretation of results, and masking of important phenomena. This study resolves various contradictory paradigms that contributed to the dogma that *P*. *aeruginosa* is exclusively an extracellular pathogen despite evidence to the contrary. The outcomes also illustrate the value of quantitative imaging to allow simultaneous spatial and temporal monitoring of bacteria and host cells to study their interactions and their respective viability when those interactions are complex and lack synchrony. In doing so they also highlight the importance of studying individual cells during infection, in addition to collecting population-based data which can potentially mask key events.

**Fig 11:**
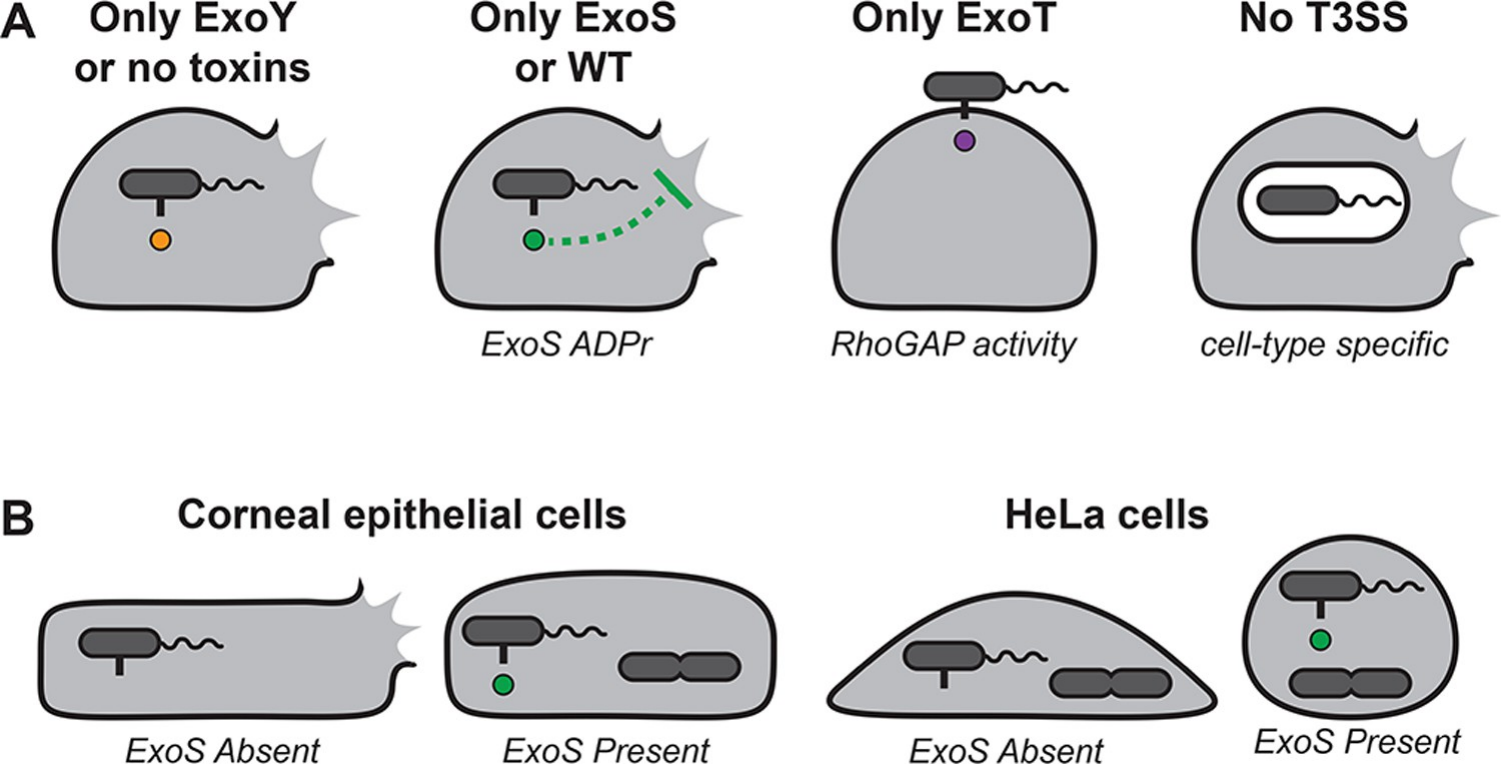
**(A)** Diagram depicting the outcomes on host cells of expressing individual T3SS effectors showing: 1) Whether bacteria are most likely to become cytoplasmic, vacuolar, or remain extracellular, and 2) whether these conditions lead to lytic death of host cells. **(B)** Diagram depicting the same outcomes as above relating to ExoS expression in corneal epithelial cells or HeLa cells.

We and others have studied roles for ExoS ADPr activity in vacuolar escape [40,43], evasion of autophagy [44], and construction of intracellular niches within plasma membrane blebs that contribute to bacterial egress [35,36]. Here, we report two more roles for ExoS ADPr activity; 1) intracellular secretion to delay cell death which otherwise destroys the protected replicative niche, and 2) suppression of the antiphagocytic RhoGAP activities of both ExoS and ExoT to allow invasion to occur (see Fig 11 for schematic overview). This helps resolve the conundrum of how invasive strains of *P*. *aeruginosa* invade cells while encoding potentially antiphagocytic effectors. More research will be required to elucidate the mechanistic details of host cell death following bacterial internalization, how ExoS attenuates cell death, and the contribution of both phenomena to the pathogenesis of infection. The results of this study provide evidence that both invasion and intracellular replication occur in cells that are viable, that antibiotic exclusion assays combined with imaging can effectively distinguish between intracellular bacteria and antibiotic resistant extracellular bacteria, and that T3SS-dependent intracellular pathogenesis can be driven by intracellular bacteria. Taken together, the results of this and previous studies suggest that a key function of ExoS is to enable *P*. *aeruginosa* to adopt an intracellular lifestyle. How *P*. *aeruginosa* balances this with its extracellular roles, some also dependent on the T3SS, remains to be determined.

## Materials and methods

### Bacterial strains and plasmids

*P*. *aeruginosa* strain PAO1 and isogenic T3SS mutants were provided by Dr. Arne Rietsch (Case Western Reserve University) [54,67]. The T3SS-GFP reporter pJNE05 and pUC18 Tn7 Prha^exsA^ were provided by Dr. Timothy Yahr (University of Iowa) [46]. Exogenous expression of ExoS and ExoT was accomplished with pUCP18 vectors provided by Dr. Joseph Barbieri (Medical College of Wisconsin). Mutations made in ExoT for RhoGAP activity were R149K, and for ADPr activity E383D/E385D [22]. ExoS mutations made for RhoGAP activity were R146K, and for ADPR activity E379D/E381D [26]. Chromosomal integration was achieved with plasmids pTNS3 and pFLP2 [68]. *P*. *aeruginosa* was transformed with plasmids by electroporation: 5 ml early log-phase cultures were washed in 300 mM sucrose three times, and 50 μl of the culture suspended in 300 mM sucrose, combined with 100 ng plasmid in a 0.2 cm gap cuvette and pulsed (200 ohms, 25 μF, 2.5 kV) for 2 seconds, followed by 1 h growth in LB media and plating on selective Dicfo Tryptic Soy Agar (TSA, BD Biosciences) plates (300 μg/ml carbenicillin or 100 μg/ml gentamicin). Chromosomal integration for rhamnose-inducible *exsA* strains was achieved by electroporation of pUC18 Tn7 Prha^exsA^ and pTNS3 simultaneously, using the same parameters as above, and plating on Vogel-Bonner media (VBM) with 80 μg/ml gentamicin. Purified colonies were grown in LB media, electroporated with pFLP2, and plated on VBM media with 300 μg/ml carbenicillin. Colonies were purified and inoculated onto solid media containing only yeast extract and tryptone with 10% sucrose. Colonies were patched onto VBM, VBM with 80 μg/ml gentamicin, and VBM with 300 μg/ml carbenicillin. Colonies that only grew on VBM were verified for rhamnose induction of the T3SS.

### Cell culture

HeLa cells were cultured in DMEM (Gibco) plus 10% fetal bovine serum (Atlanta Biologicals). For live imaging experiments, HeLa cells were seeded onto 8-well Ibitreat coated polymer micro-slides (Ibidi). Human telomerase-immortalized corneal epithelial cells (hTCEpi) were provided by Dr. Daniel Robertson (University of Texas Southwestern) [69] and were maintained in KGM-2 media (0.15 mM calcium) (Lonza) in an undifferentiated state. For differentiation, hTCEpi were exposed to KGM-2 containing 1.15 mM calcium. Differentiated cells were grown on No. 1.5 uncoated glass-bottom 24-well dishes (MatTek) for imaging experiments. The corneal cells were seeded at ~ 70% confluence to enhance *P*. *aeruginosa* internalization *via* basolateral surfaces [70].

### Infection experiments

*P*. *aeruginosa* was grown on TSA with selective antibiotics one day before experiments (37 C, 24 h). Bacteria were removed from the plate with a sterile loop and suspended in sterile PBS by gentle pipetting. MOIs were calculated using A540 of 1 = 4 x 10^8^ CFU, and bacteria were added to cells containing complete media. At 3 h post-infection, media was replaced containing 200 μg/ml gentamicin to kill extracellular bacteria. Alternatively, 200 μg/ml amikacin was used to kill extracellular bacteria during imaging experiments since GFP-expression plasmids used conferred gentamicin resistance. For enumerating internal CFU, cells were lysed by 0.25% vol/vol Triton X-100 in PBS and diluted; aliquots of each dilution series were plated onto MacConkey agar (BD Biosciences) for viable counting. The inoculum was confirmed by the same method.

### Imaging

Images were captured on a Nikon Ti-E inverted wide-field fluorescence microscope equipped with Lumencor SpectraX illumination source, or a Nikon Ti2-E with X-Cite XYLIS Broad Spectrum LED Illumination System. For live images, cultures slides were placed in an Okolab Uno-combined controller stage top incubation chamber to maintain heat, humidity, and 5% CO2. Time-lapse images were captured using a CFI Plan Apo Lambda 40X NA 0.95 air objective, or a CFI Plan Apo Lambda 20X NA 0.75 air objective, each equipped with differential interference contrast (DIC). Hoechst (Immunochemistry, 3 μl/ml) was added to aid in automated counting of cells by visualizing nuclei, and Propidium Iodide (Immunochemistry, 3 μl/ml) was used to identify permeabilized cells. Focal planes were maintained by Nikon Perfect Focus hardware. For time-lapse, fields were chosen using DIC in order to identify areas free of debris; GFP was not observed until the time-lapse was completed in order to avoid field selection bias. Eight fields were imaged for each condition leading to totals of 300–500 cells analyzed per well.

### Image analysis

Time-lapse images were computationally analyzed using a custom macro written for the FIJI package of Image J version 1.53c with the final results table encoded as intensity values in a TIF image [71]. The code for this analysis has been made available in a GitHub repository https://github.com/Llamero/Nuclei_analysis-macro. Rates of cell death for the whole field were determined by first segmenting nuclei by manually thresholding both the Hoechst and PI channels and applying a watershed to the resulting mask. A Sobel edge detector (“Find Edges”) was then used to remove out of focus (detached) nuclei from the analysis. Finally, cell death was measured as the ratio of Hoechst positive nuclei that also contained PI.

The analysis method for tracking invasion state of individual cells (Fig 5) segmented nuclei using a manually set threshold for the Hoechst channel and then tracking the ultimate points of each nucleus throughout all frames using the plugin MTrack2, generating a marker ID map of nuclei found in all frames. This map was then used to segment and track each nucleus using a marker-controlled watershed [72] and writing the results to a TIF file with one row per nucleus. To designate the cytoplasm around each nucleus, a Voronoi mask was then used to segment the local neighborhood surrounding each nucleus. Due to the asymmetrical shape of many of the cells, the Voronoi segmentation did not always correctly segment along cell boundaries, especially at points distant from the cell nucleus. To resolve this issue, we empirically capped the maximum distance for our analysis at 9 μm (50 pixels) from the cell nucleus. Background signal in the GFP channel was removed using a high-pass filter. The area, max, and median GFP intensity values for each nucleus ROI was measured and reported in the TIF results table.

TIF results images were then converted to tab-delimited results tables, assembled into data frames, and analyzed for cell death timing, invasion status, and summed GFP area using Python 3 version 3.8.5 and Pandas version 1.1.3 [73,74]. Values to designate a cut-off for Hoechst to PI ratio, GFP area minimum, and maximum intensity above background were determined empirically by viewing images from each data set, and absolute numbers varied based on acquisition parameters. This analysis code is available in the following GitHub repository; https://github.com/abbykroken/cell_survival_with_bacteria.

### Biotinylated APD ribosylation assay

Methods were adapted from Riese et al. (2002) [33]. Cells were infected with bacteria for 4 hours, and then cooled to 4°C with cold HGI buffer (20 mM PIPES, 2 mM Na-ATP, 4.8 mM magnesium acetate, 0.15 M potassium glutamate, 2 mM EGTA, and potassium hydroxide adjusted to pH 7.0, used within 1 week of making.) Tetanolysin (List Biologicals) at 200ng/mL was added in cold HGI for 20 m at 4°C. After removal of tetanolysin solution, HGI warmed to 37°C containing 4 μM biotinylated NAD (Trevigen) was added and cells incubated at 37°C for 40 minutes. Solution was removed and cells were lysed in RIPA buffer (Thermo Fisher Scientific). Nuclei and insoluble material were removed with centrifugation at 1000xg for 5 m. Samples were resolved in Laemmli buffer on 4–20% gradient Tris Glycine gels (Bio-Rad) with stain free label. Total protein was visualized in gel on a Bio-Rad ChemiDoc instrument prior- and post-transfer to nitrocellulose membrane. HA tag detected with 3F10 rat anti-HA IgG antibody (Sigma-Aldrich, 10 μg/ml) in 3% BSA (Calbiochem) in TBST (20mM Tris, 150mM NaCl, 0.1% Tween-20, pH 7.5), and secondary goat anti-rat IgG Alexa 647 antibody (Thermo Fisher Scientific, 1:1000).

### Growth and reporter assays

A 96 well plate was prepared with 150 μl TSB liquid media supplemented with 100 mM glycine and 1% glycerol. In some wells, either 0.2% rhamnose or 2 mM EGTA was included. Approximately 10^6^ bacteria transformed with the T3SS-GFP reporter pJNE05 were added to each well. A BioTek Synergy HTX multimode reader was used to incubate the plate at 37°C and read absorbance at 540 nm and GFP intensity every 15 min. The plate was shaken linearly in between readings and control conditions included non-inoculated media. Data were averaged over three technical replicates. The experiment was repeated twice, and one replicate is shown.

### Statistics

Statistical analyses were performed, and data presented using Graph Pad Prism 9. Data were shown as a mean ± standard deviation (SD) of at least three independent experiments unless otherwise indicated. Comparison of two groups was performed by Student’s t-Test, three or more groups by One-way ANOVA with Tukey’s post-hoc analysis. Non-parametric data were shown as a median with inter-quartile range (IQR) for each group and two groups compared with the Mann-Whitney U test, three or more groups using the Kruskal-Wallis test with Dunn’s multiple comparisons. A non-parametric Kolmogorov Smirnov (KS) test was performed against the PAO1 condition for complementation experiments. In each instance, * P < 0.05, ** P < 0.01, *** P < 0.005 and **** P < 0.001.

## Supporting information

S1 Video Nuclei labeling of host cells with bacterial-associated cell death. Corneal epithelial cells or HeLa cells were labeled with Hoechst (blue) and infected with wild-type PAO1 or PAO1Δ*exoSTY* and extracellular bacteria were eliminated with amikacin after 3 h. Propidium iodide (red) was included in the media to detect cell membrane permeabilization (cell death) as it occurred. Images were captured once per hour.
(MP4)

S2 Video *P*. *aeruginosa* intracellular replication and induction of host cell death. Corneal epithelial cells and HeLa cells were infected with wild-type PAO1 or PAO1Δ*exoSTY* expressing a T3SS GFP reporter (green), and extracellular bacteria were eliminated with amikacin after 3 h. Propidium iodide (red) in the media detected when cell membrane permeabilization (cell death) occurred. Images were captured once per hour.
(MP4)

S3 Video *P*. *aeruginosa* intracellular replication up to 6 h post-infection. Corneal epithelial cells were infected with wild-type PAO1 or PAO1Δ*exoSTY* expressing a T3SS GFP reporter (green), and extracellular bacteria were eliminated with amikacin after 2 h 45 m. Hoechst detected all nuclei, and propidium iodide (red) in the media detected when cell membrane permeabilization (cell death) occurred. Images were captured every 5 min from 3 to 6 h post-infection.
(MP4)

S4 Video Intracellular replication by *P*. *aeruginosa* PAO1 and a T3SS effector mutant only expressing ExoS in conjunction with host cell death. Corneal epithelial cells were infected with PAO1 or T3SS mutants missing 2 or 3 effectors for 3 h. Bacteria were transformed with the T3SS-GFP reporter (green). Extracellular bacteria were eliminated with amikacin at 3 h post-infection. Hoechst detected all nuclei, and propidium iodide was included in the media to detect when cell membrane permeabilization occurred. Time-lapse images were captured once per hour from 4 to 20 h post-infection. None of the effector mutants replicated similarly to PAO1 except those expressing only ExoS.
(MP4)

S5 Video Movies showing outcomes of rhamnose induction of T3SS expression. Corneal epithelial cells were infected with wild-type PAO1, PAO1Δ*exsA*, or PAO1Δ*exsA* Prha^exsA^ for 3 h. Bacteria were transformed with the T3SS-GFP reporter. Extracellular bacteria were eliminated with amikacin at 3 h post infection. At 3.5 h post-infection, 0.2% rhamnose was added (+rha) where indicated to induce the T3SS in the intracellular bacteria, and PI included in the media. Time-lapse images were captured once per hour from 4 to 20 h post infection. Three example fields each of PAO1 and PAO1Δ*exsA* Prha^exsA^ with rhamnose induction are shown.
(MP4)

S1 Fig HeLa cells become too fragile for gentamicin protection assays. HeLa cells were cultured in plastic tissue culture plates and infected using fluorescent *P*. *aeruginosa* PAO1-GFP or PAO1Δ*exoSTY* and media containing Propidium Iodide. Cells were imaged at indicated time points prior to and post-washing, as typically conducted in gentamicin protection assay, to show the impact of washing on cell retention. Considerable host cell loss occurs after 8 h with PAO1 but not PAO1Δ*exoSTY*.
(TIF)
