# Supplementary figures and images for: Exotoxin S secreted by internalized Pseudomonas aeruginosa delays lytic host cell death
Source: PLoS Pathog. 2022 Feb 7;18(2):e1010306. doi: 10.1371/journal.ppat.1010306 (PMC8853526; doi:10.1371/journal.ppat.1010306)

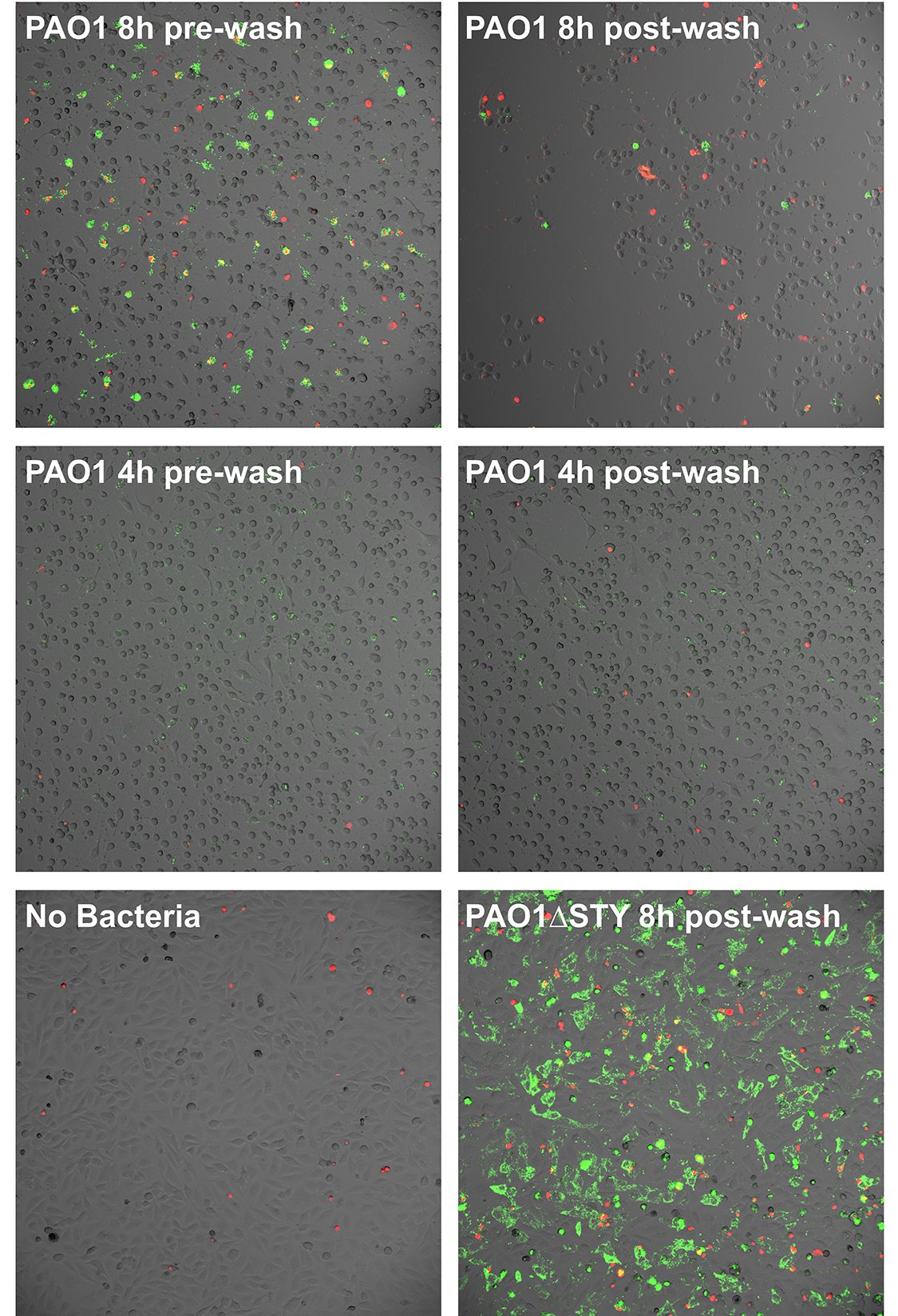

Supplement: S1 Fig — HeLa cells were cultured in plastic tissue culture plates and infected using fluorescent P. aeruginosa PAO1-GFP or PAO1ΔexoSTY and media containing Propidium Iodide. Cells were imaged at indicated time points prior to and post-washing, as typically conducted in gentamicin protection assay, to show the impact of washing on cell retention. Considerable host cell loss occurs after 8 h with PAO1 but not PAO1ΔexoSTY. (TIF) [file ppat.1010306.s006.tif]
